# Validity and performance of the Functional Assessment of Cancer Therapy-Bladder (FACT-Bl) among advanced urothelial cancer patients

**DOI:** 10.1007/s00520-019-04709-0

**Published:** 2019-03-01

**Authors:** Arnold Degboe, Cristina Ivanescu, Jeffrey M. Rohay, Ralph R. Turner, David Cella

**Affiliations:** 1 AstraZeneca, One MedImmune Way, Gaithersburg, MD 20878 USA; 2 IQVIA, Diane Bldg., Office 314, Herikerbergweg 181, 1101 CN Amsterdam-Zuidoost, The Netherlands; 3 IQVIA, 26th Floor, 485 Lexington Ave., New York, NY 10017 USA; 4 Scotch Mountain Associates, LLC, 13248 Fieldstone Way, Gainesvillle, VA 20155 USA; 5 Feinberg School of Medicine, Northwestern University, 633 N. Saint Clair St., 19th Floor, Chicago, IL 60611 USA

**Keywords:** Urinary bladder neoplasms, Patient-reported outcome measures, Health-related quality of life, Validation studies as topic, Psychometrics

## Abstract

**Purpose:**

The recent increase in emerging novel therapies in the bladder cancer therapeutic area has increased the need for fit-for-purpose patient-reported outcome (PRO) measures for these patients. This study evaluates the psychometric properties of the Functional Assessment of Cancer Therapy-Bladder (FACT-Bl) in 182 patients with advanced urothelial cancer (UC) and fills an important gap by demonstrating its validity for use in clinical trials.

**Methods:**

Data were collected as part of a multicentre, open-label study of durvalumab in patients with inoperable or metastatic solid tumours. Psychometric properties evaluated include item and subscale characteristics (including correlation analysis), reliability (estimated using Cronbach’s *α*), validity (by independent sample *t* test), responsiveness (using mixed models with repeated measures), and clinically meaningful changes using both anchor-based and distribution-based methods.

**Results:**

One hundred and seventy-two patients completed the FACT-Bl questionnaire at baseline. Many individual items had floor or ceiling effects indicative of minimal symptoms and high functioning. The psychometric properties of the existing established scales were assessed and found to range from acceptable to very good. Internal consistency (most Cronbach’s *α* coefficients range 0.66–0.85) and stability (test–retest reliability) generally exceeded standards for good reliability (most estimated intraclass correlations [ICCs] exceeded 0.70, although ICCs for some items [e.g. emotional well-being, ICC 0.58; social well-being, ICC 0.66] were lower than 0.70). Evidence for known-group validity of relevant FACT-Bl subscales and total score was demonstrated by significant differences between groups defined by baseline tumour burden and quality of life scores (difference of FACT-Bl total between low/high tumour burden groups 11.72 (*p* = 0.001); difference between low/high QoL scores groups 30.51 (*p* < 0.0001)). The FACT-Bl subscale and total scores were responsive to changes in bladder cancer symptom severity. Clinically meaningful changes in FACT-Bl scores were estimated.

**Conclusions:**

Results support the use of the FACT-Bl within this patient population in future clinical research.

## Introduction

In 2018, there were approximately 549,000 new cases of bladder cancer worldwide, and bladder cancer accounted for approximately 200,000 deaths [1]. The vast majority of bladder cancer cases (90%) were urothelial carcinoma [2, 3].

The burden of advanced urothelial cancer (UC) is attributable to disease and treatment characteristics [4, 5]. Haematuria, urinary frequency and urgency, and pain are among the most common signs and symptoms [6]. Additionally, symptoms such as bleeding, pain, dysuria, constipation, fatigue, emotional distress, and urinary obstruction adversely impact QoL in advanced bladder cancer [5]. Treatment-related side effects of fatigue, and the impact on daily activities, are also reported as relevant to these patients [5], as well as issues with self-esteem, embarrassment, and difficulty engaging in sexual relationships [4, 7, 8]. Emerging novel treatments [9] have accelerated interest in developing and validating patient-reported outcome (PRO) collection instruments to gain a full understanding of UC and disease impact, information important for patients and clinicians. In addition, PRO data can inform the benefit/risk assessment for regulators and payers [10].

Many instruments exist to assess health-related quality-of-life (HRQoL) in UC [11, 12]. PRO data are particularly important for these patients due to the burden of disease and therapy [11]. The Functional Assessment of Cancer Therapy-Bladder (FACT-Bl) has been used in several studies, often to determine comparative effects of various interventions on HRQoL [6–8, 13–16]. Despite its wide use, validity data in patients with locally advanced or metastatic UC are not published. The current study confirms the validity of the FACT-Bl in patients with urothelial carcinoma and follows an approach that is consistent with FDA guidelines for PRO validation [17]. It assesses the psychometric properties of the FACT-Bl in a group of patients who participated in the combined phase 1/2 clinical trial of durvalumab monotherapy. Funding for this research was provided by AstraZeneca.

## Materials and methods

### Study design

A multicentre, open-label (dose-escalation, dose-exploration, and dose-expansion) study was previously conducted to evaluate durvalumab’s safety, tolerability, and antitumour activity in patients with inoperable or metastatic solid tumours (Study 1108; NCT01693562). Study results are published elsewhere [18]. A total of 182 patients with upper and lower tract UC who received and have progressed or are refractory to 1 or 2 prior lines of systemic therapy for inoperable or metastatic disease, including a standard platinum-based regimen, were included in that open-label study. That clinical study was conducted according to the Declaration of Helsinki and approved by the independent ethics committee or institutional review board at each participating centre, with written informed consent obtained from all patients.

### Data collection

PROs were evaluated using pen-and-paper versions of the FACT-Bl, the European Organisation for Research and Treatment of Cancer Quality-of-Life Questionnaire C30 (EORTC QLQ-C30), and a one-item pain questionnaire [19] completed at the screening visit: on day 1 of treatment doses 1, 3, and 5; at weeks 6, 12, and 16; and every 8 weeks until end of treatment (12 months).

### Measures

#### FACT-Bl

The FACT-Bl (version 4) is a multidimensional, self-administered 39-item questionnaire to assess patient bladder cancer-specific symptoms using a ‘core’ set of questions (Functional Assessment of Cancer Therapy-General; FACT-G), a cancer site-specific bladder subscale, and HRQoL [20, 21]. Table 1 summarises the five subscales and three summary scores produced by the FACT-Bl.

**Table 1:** Functional Assessment of Cancer Therapy-Bladder Cancer (FACT-Bl) subscales, summary, and prioritised item characteristics and scores at baseline

Scale	Description	No. of items	Score range	Number	Mean	STD	Median	Min	Max
PWB	Physical well-being	7	0–28	172	20.4	6.3	22.0	2.0	28.0
SWB	Social/family well-being	7	0–28	172	22.2	5.1	23.3	3.0	28.0
EWB	Emotional well-being	6	0–24	171	17.3	4.3	18.0	4.0	24.0
FWB	Functional well-being	7	0–28	171	15.8	6.5	16.0	0.0	28.0
BlCS	Bladder cancer subscale	12	0–48	169	31.9	7.4	33.3	15.6	48.0
TOI	Trial Outcome Index	26	0–108	169	68.0	17.4	69.6	30.0	104.0
FACTG	FACT-G total score	27	0–108	171	75.6	17.5	77.2	21.7	108.0
TOTAL	FACT-Bl total score	39	0–156	169	107.5	23.0	107.2	45.7	156.0
NFBlSI-18	NCCN-FACT Bladder Symptom Index-18	18	0–72	172	47.7	13.3	49.5	13.5	72.0
NFBlSI-DRS-P	NCCN-FACT Bladder Symptom Index Disease Related Symptoms-Physical	9	0–36	171	22.7	7.3	24.4	4.5	36.0
GP1	Prioritised fatigue item (‘I have a lack of energy’)	1	0–4	172	2.3	1.2	2	0	4
GP4	Prioritised pain item (‘I have pain’)	1	0–4	172	2.4	1.3	3	0	4

#### NFBISI-18

The National Comprehensive Cancer Network FACT Bladder Symptom Index (NFBlSI-18), a measure of advanced bladder cancer-specific symptoms, assesses symptoms perceived as most important by patients and oncology clinical experts. The NFBISI-18 is based almost entirely on the FACT-Bl, including 16 items from the FACT-Bl instrument plus two items that have not been previously included (‘I feel weak all over’ and ‘I feel light-headed [dizzy]’) [5]. These two items were added based on qualitative analysis of patient and clinician priorities for symptoms and concerns associated with receiving treatment for advanced bladder cancer.

NFBlSI-18 yields three subscale scores (i.e. disease-related symptoms, treatment side effects, and general function/well-being) and a summary score. Items are rated on a 5-point Likert scale ranging from 0 = ‘not at all’ to 4 = ‘very much’ with a 7-day recall period. Higher scores represent better QoL. The subscale and summary scores are calculated using the Manual of Functional Assessment of Chronic Illness Therapy (FACIT) Measurement System [22]. Only the NFBlSI-18 total summary score and the disease-related symptoms-physical subscale (NFBlSI-DRS-P) score are considered in the current analyses and prorated based on the 16 available items.

### Statistical analysis

Psychometric analyses were performed on the full analysis set population using baseline (dose 1, day 1) data only, except for test–retest which used dose 3, day 29 and the responsiveness analysis where data up to dose 7, day 85 were used as period 2.

#### FACT-Bl item and scale characteristics

Performance of the 39 FACT-Bl items was evaluated by means of descriptive statistics (mean, standard deviation) and percentage of lowest and highest responses (floor and ceiling effects, respectively) at baseline (dose 1, day 1). Single items regarding pain and fatigue were prioritised in the clinical trial and examined specifically in psychometric analyses. Characteristics of FACT-Bl subscales for physical, functional, social/family, and emotional well-being and the Bladder Cancer subscale (PWB, FWB, SWB, EWB, and BlCS, respectively) as well as total summary scores (FACT-G total score, FACT-Bl total score, FACT-l Trial Outcome Index [TOI], and NFBlSI-18) were summarised using measures of central tendency (e.g. mean, median) and variability (e.g. standard deviation [SD], interquartile range).

#### Correlation analysis

Item-to-item, item-to-total, and between-scales correlations were assessed using Spearman correlation coefficients.

#### Reliability

Internal consistency reliability of subscale and total scores was estimated using Cronbach’s *α*. To evaluate reliability in stable patients, a group of patients whose EORTC-C30 QoL score was within ± 0.25 standard deviations of their baseline score was isolated. We evaluated intraclass correlation coefficients (ICCs) between baseline (period 1) and dose 3 (day 29) (period 2) [23]. Coefficients of 0.6 and higher are considered acceptable, and coefficients of 0.7 and higher are considered good [23].

#### Construct validity

Construct validity testing included convergent validity and known-group validity. Convergent validity was assessed at baseline using a Spearman correlation with EORTC QLQ-C30 domain scores. Known-group validity was examined by independent sample *t* test comparing baseline mean FACT-Bl scale scores by baseline tumour burden (above and below the median value, i.e. 59.9 mm) and by the baseline EORTC QLQ C-30 global health status/QoL score (above and below the median value, i.e. 50 points).

#### Responsiveness

The ability to detect change was assessed by comparing changes in the FACT-Bl scores over time between responders and non-responders using mixed models with repeated measures. Assessments up to and including dose 7 (day 85) were included in the analysis to maximise the longitudinal window and ensure sufficient sample size. Responders versus non-responders were defined in two ways: objective response (responders defined as patients with a confirmed objective complete or partial tumour response [18]; non-responders included the remainder of the patients [*n* = 150]) and patient evaluation of change using global health status (GHS)/QoL (patients demonstrating at least a 10-point improvement in GHS/QoL scale at dose 7 (day 85) compared with their baseline score were classified as responders and the remainder of patients as non-responders). The FACT-Bl was considered responsive if the mean change from baseline to dose 7 (day 85) is > 0 and statistically significant (indicating improvement) for the responder group and < 0 and statistically significant (indicating deterioration) for non-responders.

#### Clinically meaningful thresholds

As in other published studies [24], both anchor-based and distribution-based methods were used to explore a preliminary clinically meaningful change (CMC). For anchor-based methods, two external anchors using the objective response based on the Response Evaluation Criteria in Solid Tumours (RECIST) [25] criteria and the EORTC-C30 GHS/QoL scale at day 57 were used.

Several alternative methods were tested for convergence on the CMC using a robust sample size. Day 57 was selected as the most distant time point with at least 50% of the patients with a baseline reporting a score. The external anchors were as follows: (1) patients with objective response classified as ‘responders’, and the remaining patients as ‘non-responders’ and (2) patients classified as GHS/QoL responders/non-responders using the established clinical meaningful threshold of 10 points (as described above). Three distribution-based methods were also used: (1) a 0.5 SD, (2) 1 standard error of measurement (SEM) at visit baseline, and (3) reliable change index. *T* tests were performed to compare mean changes from baseline for clinical responders versus non-responders.

All data preparation and analyses were performed using SAS version 9.3 (SAS Institute, NC) or higher. Statistical comparisons were made using two-sided tests at *α* = 0.05 significance level unless specifically stated otherwise. Due to the exploratory nature of the analyses, adjustments for multiple comparisons were not made.

All analyses, except for item characteristics, were performed on items recoded as necessary with higher scores indicating better QoL.

## Results

### Baseline demographics and patient response

As of data cut-off (24 October 2016), 191 patients were treated for locally advanced or metastatic UC, 182 of which had progressed after platinum-based therapy [16]. Table 2 provides detailed patient demographics. Further demographic details are published elsewhere [18].

**Table 2:** Patient baseline demographics

Patient characteristics	2L+ post-platinum UC (*N* = 182)
Age, median (range), years, *n* (%)	67.0 (34–88)
Sex, *n* (%)	
No.	182
Female	51 (28.0)
Male	131 (72.0)
Race, *n* (%)	(*n* = 165)
Asian	15 (17.4)
Black/African-American	4 (4.7)
White	65 (75.6)
Other	2 (2.3)
ECOG performance status, *n* (%)	
0	61 (33.5)
1	121 (66.5)
Stage 4 at study entry, *n* (%)	182 (100)
Sites of disease at baseline^†^	
Visceral	168 (92.3)
Liver	78 (42.9)
Lymph nodes only	14 (7.7)

Further details on patient demographics are published elsewhere [16]

*ECOG* Eastern Cooperative Oncology Group

†Site of disease at baseline was derived from the baseline disease assessment by the investigator and blinded independent central review (BICR). Visceral metastases defined as liver, lung, bone, or any non-lymph node or soft-tissue metastases

### Questionnaire completion/compliance

Out of 182 patients in the second-line-or-later (2L+) post-platinum UC subgroup, 172 (94.5%) completed the FACT-Bl questionnaire at baseline. Response rate was high (over 92%) for all items. Two questions were for patients with ostomies only (46 [26%] patients answered these questions). Two questions about sexuality were asked: one for all patients (89 [49%] patients responded) and one for men only (65 [36%] patients responded). Compliance specific to eligible populations for these questions was not calculated.

### Item and scale performance

Subject responses covered the entire range (0–4) for each FACT-Bl item. The majority of items had floor or ceiling effects reflecting minimal symptoms and high functioning. Issues were noted for the three items addressing sexual functioning where at least 25% of the patients reported the lowest response option.

Mean values for FACT-Bl and FACT-G total were 107.5 (range 45.7–156.0) and 75.6 (range 21.7–108.0), respectively, which represent 69% and 70%, respectively, of the scale range. The mean score for the FACT-Bl FWB scale was 15.8 (range 0.0–28.0), representing moderately impaired functioning. Subscales and summary scores at baseline are reported in Table 1.

### Correlation analysis

The patterns of correlations matched expectations, with items in a scale correlating more highly with the score of that scale than with the score of other scales in the instrument, and all subscales correlating strongly with the FACT-Bl total score and FACT-G total score. The highest and second highest correlation coefficient was 0.91 observed between FWB and FACT-G and 0.81 observed between the PWB and FACT-G, respectively. All subscales correlated moderately or higher with the TOI or NFBlSI-18 index. The SWB subscale had very low correlations (*r* < 0.3) with the PWB subscale and moderate correlation with EWB and BlCS subscales. Table 3 shows the subscale and summary score correlations.

**Table 3:**
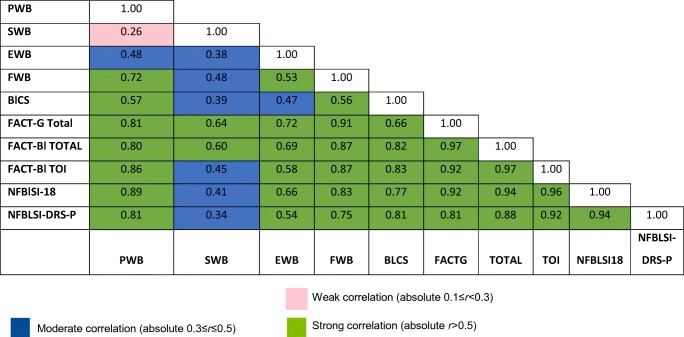
Between subscale and summary score correlations

### Reliability analysis

All subscales and summary scores demonstrate adequate to good internal consistency (Cronbach’s *α* range 0.63 to 0.93). The BlCS subscale internal consistency (Cronbach’s *α* value of 0.63) was slightly lower than the generally recommended 0.70 [26]. Composed of different symptoms, the BlCS subscale demonstrated more inter-item variability across patients.

Minimal change in the mean subscale or mean summary scores from baseline to dose 3 (day 29) demonstrated good test–retest reliability. The estimated ICC for the two visits (4 weeks apart) ranged from 0.58 (EWB) to 0.80 (FACT-G total score), with the lower bound of the 95% CI ranging from 0.45 (for EWB) to 0.85 (for FACT-G total score). All ICCs exceeded 0.70, except for emotional well-being (ICC 0.58) and social well-being (ICC 0.66). The mean ICC was 0.72. This confirms that FACT-Bl and the NFBlSI-18 showed fair to very good reliability for all dimensions in this patient population. The single items ‘I have a lack of energy’ and ‘I have pain’ also demonstrate acceptable reliability with ICC values of 0.60 and 0.70, respectively.

### Construct validity

As could be expected, the physical functional domain from EORTC-QLQ C30 correlates highly (*r* ≥ 0.77) with the PWB. Strong correlations are observed also with the summary scores (FACT-BL total score, FACT-G total score, TOI and NFBLSI-18) and the FWB and BLCS. The EORTC-QLQ C30 emotional functional domain correlates highly (*r* ≥ 0.72) with EWB. In contrast, the EORTC-QLQ C30 social functional domain correlates weakly with SWB (*r* = 0.22).

The fatigue domain from EORTC-QLQ C30 correlates highly with the single-item GP1 (fatigue) from FACT-BL (*r* = − 0.8), and the pain domain from EORTC-QLQ C30 correlates highly with the single-item GP4 (pain) from FACT-BL (*r* = − 0.9). Note that the negative value of the Spearman correlation coefficient is due to the fact that higher scores on the fatigue and pain domains from EORTC-QLQ C30 indicate a worse health state while higher values on the single-items GP1 (fatigue) and GP4 (pain) indicate better health state.

The EORTC QLQ-C30 global health status/QoL score from EORTC-QLQ C30 correlates highly with FACT-G, FACT-TOI, FACT-BL total score, and NFBISI-18 (*r* > 0.76). Evidence for known-group validity was found for the FACT-Bl and NFBlSI-18 through significant differences between groups defined by baseline tumour burden and EORTC QLQ C-30 health status/QoL scores (Table 4). Increased tumour burden was associated with lower scores (worse health status and more symptoms). This finding holds for all the investigated scores except for the SWB and EWB where the difference between groups was not significant. Similarly, the FACT-Bl could show significant differences between groups defined by the GHS/QoL scores at baseline.

**Table 4:** Mean score at baseline by tumour burden and EORTC QLQ C-30 GHS/QoL

Scale/item	Scale/item description	Tumour burden	*N*	Mean	SD	*P* value	EORTC QLQ C-30 GHS/ QoL	*N*	Mean	SD	*P* value
GP1	I have a lack of energy	< Median (59.9 mm)	84	2.60	1.16		≤ Median (50 points)	52	1.67	1.17	
		≥ Median (59.9 mm)	83	1.99	1.26		> Median (50 points)	53	2.77	1.09	
		Difference		0.61	1.21	0.0015	Difference		− 1.10	1.13	< 0.0001
GP4	I have pain	< Median (59.9 mm)	84	2.75	1.29		≤ Median (50 points)	52	1.77	1.32	
		≥ Median (59.9 mm)	83	2.06	1.31		> Median (50 points)	53	3.00	1.11	
		Difference		0.69	1.30	0.0008	Difference		− 1.23	1.22	< 0.0001
PWB	Physical well-being	< Median (59.9 mm)	84	22.26	5.60		≤ Median (50 points)	52	16.16	6.21	
		≥ Median (59.9 mm)	83	18.44	6.49		> Median (50 points)	53	23.75	4.03	
		Difference		3.82	6.06	< 0.0001	Difference		− 7.58	5.22	< 0.0001
SWB	Social/family well-being	< Median (59.9 mm)	84	22.69	4.93		≤ Median (50 points)	52	19.70	5.32	
		≥ Median (59.9 mm)	83	21.62	4.96		> Median (50 points)	53	22.79	5.21	
		Difference		1.06	4.94	0.1662	Difference		− 3.10	5.26	0.0032
EWB	Emotional well-being	< Median (59.9 mm)	83	17.67	4.29		≤ Median (50 points)	52	14.98	4.20	
		≥ Median (59.9 mm)	83	16.88	4.32		> Median (50 points)	52	19.43	3.66	
		Difference		0.78	4.31	0.2445	Difference		− 4.45	3.94	< 0.0001
FWB	Functional well-being	< Median (59.9 mm)	83	17.24	6.44		≤ Median (50 points)	52	11.93	4.80	
		≥ Median (59.9 mm)	83	14.08	6.19		> Median (50 points)	52	18.18	5.93	
		Difference		3.16	6.32	0.0015	Difference		− 6.25	5.39	< 0.0001
BlCS	Bladder cancer subscale	< Median (59.9 mm)	82	33.32	7.14		≤ Median (50 points)	51	26.88	6.27	
		≥ Median (59.9 mm)	82	30.42	7.47		> Median (50 points)	52	35.60	6.38	
		Difference		2.89	7.31	0.0122	Difference		− 8.71	6.33	< 0.0001
FACT-G Total	FACT-G total score	< Median (59.9 mm)	83	79.85	16.37		≤ Median (50 points)	52	62.77	14.70	
		≥ Median (59.9 mm)	83	71.03	17.75		> Median (50 points)	52	84.16	13.98	
		Difference		8.82	17.07	0.0011	Difference		− 21.40	14.34	< 0.0001
FACT-Bl TOI	Trial Outcome Index	< Median (59.9 mm)	82	72.66	15.88		≤ Median (50 points)	51	54.72	13.93	
		≥ Median (59.9 mm)	82	62.85	17.71		> Median (50 points)	52	77.48	13.25	
		Difference		9.81	16.82	0.0003	Difference		− 22.75	13.59	< 0.0001
FACT-Bl TOTAL	FACT-Bl total score	< Median (59.9 mm)	82	113.00	21.45		≤ Median (50 points)	51	89.25	18.09	
		≥ Median (59.9 mm)	82	101.30	23.37		> Median (50 points)	52	119.80	18.34	
		Difference		11.72	22.43	0.0010	Difference		− 30.51	18.22	< 0.0001
NFBlSI-18	NCCN-FACT Bladder Symptom Index-18	< Median (59.9 mm)	84	51.22	12.36		≤ Median (50 points)	52	37.75	10.96	
		≥ Median (59.9 mm)	83	43.79	13.58		> Median (50 points)	53	55.03	9.47	
		Difference		7.42	12.98	0.0003	Difference		− 17.28	10.24	< 0.0001
NFBlSI-DRS-P	NCCN-FACT Bladder Symptom Index Disease Related Symptoms-Physical	< Median (59.9 mm)	83	24.72	6.65		≤ Median (50 points)	52	18.06	6.64	
		≥ Median (59.9 mm)	83	20.64	7.55		> Median (50 points)	52	26.30	5.90	
		Difference		4.08	7.12	0.0003	Difference		− 8.23	6.28	< 0.0001

### Responsiveness

The FACT-Bl subscale and total scores examined in this study were responsive to changes in bladder cancer symptom severity during a 12-week time frame. The mean change from baseline to dose 7 (day 85) was > 0 (indicating improvement) for almost all FACT-Bl scores for the responder group, while estimates < 0 (indicating deterioration) were observed for non-responders, regardless of which criterion was used to define responders (objective tumour response or EORTC QLQ C-30 GHS/QoL). The mean change from baseline to dose 7 (day 85) for responders using the GHS status/QoL ranged from 0.75 (for NFBlSI-18) to 21.1 (FACT-Bl total score), compared with − 2.95 (FACT-Bl total score) to 0.06 (BlCS) for non-responders. Similarly, the mean change from baseline to dose 7 (day 85) for responders using clinical measure objective response ranged from − 1.05 (for SWB) to 12.0 (FACT-Bl total score), compared with − 3.27 (FACT-Bl total score) to 0.16 (SWB) for non-responders.

### Clinically meaningful change

The estimated clinically meaningful thresholds are provided in Table 5. Of note are the FACT-Bl total score ranges of 6.2–11.5 (rounded to 6–12), the FACT-Bl TOI of 5.4–8.7 (rounded to 5–9), the fatigue item (GP1) ranges of 0.6–1.1 (rounded to 1–2), the pain item (GP4) ranges of 0.7–1.0 (rounded to 1–2), and the NFBISI-18 ranges of 4.4–6.7 (rounded to 4–7).

**Table 5:** Clinically meaningful thresholds

Scale/item	Scale/item description	½ SD	SEM	RCI	Mean distributional methods	Anchor–clinical group		Anchor–PRO group		Mean CMT
						No. of responders	Mean	No. of responders	Mean	
GP1	I have a lack of energy	0.62	0.78	1.11	0.84	26	0.54	17	0.94	0.77
GP4	I have pain	0.66	0.72	1.02	0.80	26	0.65	17	0.94	0.80
PWB	Physical well-being	3.14	2.22	3.14	2.83	26	2.55	17	4.46	3.28
SWB	Social/family well-being	2.53	1.98	2.80	2.44	26	− 0.32	17	0.03	0.93
EWB	Emotional well-being	2.14	2.14	3.03	2.44	26	2.22	17	2.18	2.28
FWB	Functional well-being	3.25	2.24	3.17	2.88	25	3.20	17	3.71	3.27
BlCS	Bladder cancer subscale	3.70	4.51	6.38	4.86	25	3.48	16	4.15	4.10
FACT-Bl TOI	Trial Outcome Index	8.68	5.43	7.68	7.27	24	9.20	16	12.38	9.46
FACT-G Total	FACT-G total score	8.75	4.53	6.41	6.57	25	7.34	17	10.37	8.33
FACT-Bl TOTAL	FACT-Bl total score	11.51	6.18	8.75	8.81	24	10.70	16	14.18	11.26
NFBlSI-18	NCCN-FACT Bladder Symptom Index-18	6.66	4.41	6.24	5.77	26	7.97	17	11.03	8.28
NFBlSI-DRS-P	NCCN-FACT Bladder Symptom Index Disease Related Symptoms-Physical	3.65	3.92	5.54	4.37	26	4.65	17	5.73	4.92

*SD* standard deviation, *SEM* standard error of measurement, *RCI* reliable change index, *CMT* clinically meaningful thresholds (average of mean distributional methods, anchor–clinical group, and anchor–PRO group)

**Fig. 1:**
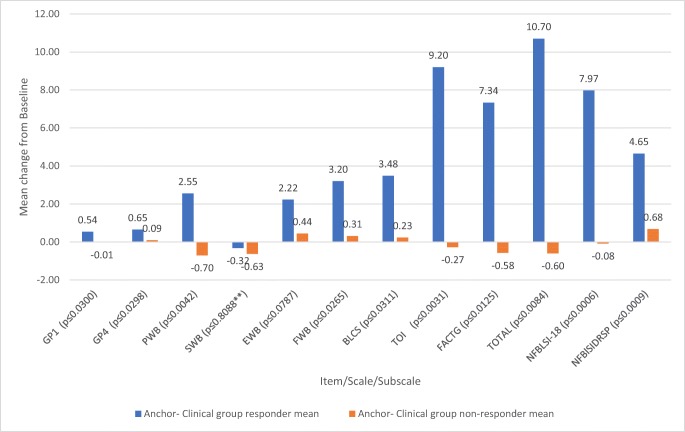
Mean score changes from baseline in clinical anchor-based responders and non-responders. Notes: Independent sample *t* test, *p* value from pooled *t* test unless otherwise noted. *p* value from Satterthwaite approximation (double asterisks)

The anchor-based estimates for clinically meaningful threshold estimates were larger and were reconciled with the distribution-based estimates to provide final estimates. Figure 1 shows the mean change for responders and non-responders in the clinical anchor group.

## Discussion

This paper reports on the psychometric properties of the FACT-Bl in patients with locally advanced or metastatic UC. Our study fills a gap in the psychometric evidence for the FACT-Bl, following FDA guidelines for PRO validation [17], and may be useful for other studies of patients with advanced UC.

Results from the UC patient cohort Study 1108 showed clinically favourable activity and an acceptable safety profile for durvalumab [18]. The current analysis showed an overall high completion of the FACT-Bl and provided additional information on outcomes important to these patients. The completion rates were above the minimum required for scoring the scales and subscales.

The psychometric properties of the existing FACT-Bl scales and the pain and fatigue items were found to be very good, with correlations in the range of others accepted throughout the validation literature [27–29]. In addition to reliability, the FACT-Bl subscale and total scores showed good evidence for construct validity and were responsive to changes in UC symptom severity during a 12-week time frame assessed by both objective tumour response and patient evaluation of change, suggesting appropriateness of the instrument to detect symptomatic change.

This study has some limitations. First, the FACT-Bl was created several years ago, and many new therapies have been developed that address symptoms, and are associated with side effects, which are not necessarily captured in the FACT-Bl. Although this questionnaire does include an overall side-effect bother item (item GP5), it is possible that additional items may be needed to assess the impact of newer treatments on patients’ lives. Second, these data were obtained from patients participating in a clinical trial, and the results may not be completely generalisable to all patients with advanced UC.

The NFBlSI-18 is an abbreviated version of the FACT-Bl that adds two new questions to the 16 FACT-Bl questions that are deemed most important by patients with advanced cancer and by clinicians who care for them [5]. In this report, the total NFBlSI-18 score was prorated based on the 16 items in the FACT-Bl. So, although these results provide support for the use of the more-focused NFBlSI-18, more data on the 18-item version will be important to more fully understand its validity.

Another limitation of the study is the unavailability of anchors (e.g. the Patient Global Impression of Change [PGIC] [30]) to determine clinically meaningful thresholds. However, very useful clinical trial end point anchors were used, including tumour response data and the scores on the EORTC QLQ C-30, another commonly used QoL questionnaire. Our analysis suggests that the established thresholds of 2–3 points for the PWB, FWB, EWB, and SWB subscales and 5–7 points for FACT-G are appropriate for this patient population [31]. These ranges are comparable to those found in studies of patients with other cancers, including the prostate [32], lung [33], and breast [31].

The ICC coefficients showed good stability of the FACT-Bl. However, the use of the 29-day post assessment as a proximal measure for test–retest reliability may have attenuated the ICC coefficients, as real change may have occurred during this period. The ICCs for EWB and SWB did not exceed the threshold of 0.70, although ICCs for these two subscales are typically lower than for other subscales, as has been demonstrated in similar studies [34].

## Conclusions

Psychometric properties of the existing established scales for the FACT-Bl as well as the NFBISI-18 were found to be very good for use in this population of advanced urothelial cancer patients. Emerging therapies for bladder cancer have accelerated interest in the development and validation of PRO instruments for this patient population to capture meaningful improvements in quality-of-life and symptom outcomes.
